# Future Challenges for Physical Therapy during and after the COVID-19 Pandemic: A Qualitative Study on the Experience of Physical Therapists in Spain

**DOI:** 10.3390/ijerph18168368

**Published:** 2021-08-07

**Authors:** Domingo Palacios-Ceña, César Fernández-de-las-Peñas, Lidiane L. Florencio, María Palacios-Ceña, Ana I. de-la-Llave-Rincón

**Affiliations:** 1 Research Group of Humanities and Qualitative Research in Health Science (Hum & QRinHS), Department of Physical Therapy, Occupational Therapy, Rehabilitation and Physical Medicine, Universidad Rey Juan Carlos, 28922 Madrid, Spain; domingo.palacios@urjc.es; 2 Research Group of Manual Therapy, Dry Needling and Therapeutic Exercise (GITM-URJC), Department of Physical Therapy, Occupational Therapy, Rehabilitation and Physical Medicine, Universidad Rey Juan Carlos, 28922 Madrid, Spain; lidiane.florencio@urjc.es (L.L.F.); maria.palacios@urjc.es (M.P.-C.); anaisabel.delallave@urjc.es (A.I.d.-l.-L.-R.)

**Keywords:** COVID-19, outbreak, physical therapy, healthcare, qualitative research

## Abstract

This qualitative exploratory study addressed the perspectives of Spanish physical therapists (PTs) regarding (a) the organization of their work during the first wave of the pandemic; (b) their role within the intensive care units (ICUs); (c) management of COVID-19 survivors; (d) potential future challenges identified for the physical therapy profession. Thirty PTs who had worked at a National Public Hospital in Madrid during the first COVID-19 outbreak were recruited by purposeful sampling and snowball techniques. In-depth interviews and researcher field notes were used to collect data. Interviews were transcribed verbatim. An inductive thematic analysis was used to identify emerging themes. After identifying 1110 codes, four themes emerged. Throughout the first wave of the pandemic, the role and work of PTs in hospitals experienced a change. These changes took place at their organizational level, affecting the distribution of PTs in the hospital, and the role of PTs in front-line COVID units such as ICUs, as well as direct management of outpatients at the onset of the pandemic, and after discharge from the ICUs. This situation has led to PTs foreseeing challenges and developing new expectations concerning their role and the physical therapy profession in the future.

## 1. Introduction

On 11 March 2020, the WHO declared a global pandemic situation for the new severe acute respiratory syndrome coronavirus 2 (SARS-COV-2) [1]. The SARS-COV-2 virus has a high risk of contagion and propagation and, hence, has overwhelmed worldwide health systems [2,3]. As a result, two-thirds of infected patients exhibit acute respiratory distress syndrome (ARDS) [4], resulting in 5% of those infected requiring admissions to an intensive care unit (ICU) for respiratory support due to respiratory failure [5]. 

To prevent a rapid spread of the SARS-COV-2 virus and to meet the demand for care, during the first outbreak, hospitals suspended all non-acute care activities, prioritizing the coverage of first-line COVID-19 treatment units [6,7,8]. Rehabilitation services were among all the services that were closed during the first wave of the pandemic, causing suspension and cancelations of treatment in inpatient/outpatient rehabilitation setting services [6,7,9,10].

In Spain, this closure of the rehabilitation services, in addition to the suspension of the treatment, resulted in the use of rehabilitation wards to create new beds for patients with COVID-19, and the redistribution of all physical therapists (PTs) to other units [11,12,13]. This situation resulted in many PTs being integrated into front-line units against COVID-19 such as ICUs without previous training [13,14]. In Spain, there are no recognized physical therapy specialties (i.e., respiratory and/or ICU), therefore, the majority of PTs working at hospital setting belong to the rehabilitation services and are not regularly integrated as part of other units such as ICU, anesthesia, pain management, pneumology, neurology, or traumatology [15].

Qualitative research helps to describe complex processes which have a great impact on healthcare professionals and healthcare organizations and can help to understand how the COVID-19 pandemic has influenced the perspective, beliefs, and attitudes of PTs [16]. Today, only three qualitative studies have exclusively described the experiences of PTs during the COVID-19 pandemic [13,14,17]. These studies had portrayed the emotional impact of PTs working in front-line units [14], their perspective on the pandemic, their experience upon arriving home [17], and ethical and professional issues that PTs have faced during their assignment to acute COVID-19 units [13].

However, to date, no study has described the perspective of PTs, during the COVID-19 outbreak, organization of their professional activity in the hospital, their integration into front-line COVID-19 units, their experience managing patients who had survived COVID-19 and how this situation could impact their future perspectives of physical therapy profession. Therefore, the purpose of this study was to explore the experiences and perspectives of Spanish PTs regarding (a) the organization of their work during the first wave of the COVID-19 pandemic; (b) their role in ICUs; (c) management of COVID-19 survivors; (d) future challenges identified for the physical therapy profession.

## 2. Materials and Methods

The current study is part of a larger qualitative research project aiming to analyze the perspective of PTs during the first outbreak of the COVID-19 pandemic. In the first study, the objective was to explore the experiences and perspectives of PTs working on the frontline in Spanish public hospitals during the COVID-19 pandemic [13]. The second study reported the emotional experience and feelings of PTs working on the frontline [14]. The current one is the last stage of the project showing future challenges in the profession due to the COVID-19 outbreak. The study followed the Standards for Reporting Qualitative Research (SRQR) [18] and Consolidated Criteria for Reporting Qualitative Research (COREQ) [19] and received approval by the Local Ethics Committee of Universidad Rey Juan Carlos (URJC 1905202011920).

### 2.1. Design

A qualitative exploratory study was conducted based on an interpretive framework [20,21,22]. The aim of an explorative descriptive qualitative study is to identify an event or a critical situation. It seeks to show “what is happening” and “how it is happening” [23]. Qualitative exploratory studies aim to be a comprehensive summary of events in the everyday terms of the described event. This design is the method of choice when straight descriptions of phenomena are desired [24,25]. On the other hand, from interpretive perspective, human action is meaningful, and the goal of inquiry is understanding how people respond and understand the meaning of social phenomena [21,22]. This method has been extensively described in the first part of the study previously published in the same journal [14]. We summarize in the current text the most relevant topics. 

### 2.2. Setting, Participants, and Sampling Strategies

The method consisted of recruiting 30 PTs using purposeful sampling (*n* = 12) and snowball sampling (*n* = 18) [22,26]. The inclusion criteria were being a PT working on the frontline (direct contact with COVID-19 patients) in public hospitals during the first wave of the COVID-19 pandemic. The recruited participants belonged to 11 public hospitals in Madrid (Spain), one of the most affected European cities. The sample size was determined according to Turner-Bowker et al. [27] who reported that 99.7% of concepts, themes, and contents emerged with 30 interviews.

### 2.3. Data Collection

Based on the qualitative exploratory design, in-depth interviews were used as the main data collection tool. During the interviews, a semi-structured question guide was used (Table 1). In addition, researcher field notes were kept during the interviews [22,26]. 

The interview guide retrieved and utilized previous knowledge on the topic to gain a comprehensive and adequate understanding of the phenomenon under study. During the interviews, researchers used prompts to resolve confusion (paraphrasing something that the participant had said) and to encourage the participant to provide further details (‘Can you tell me a bit more about that?’). This enabled relevant information to be collected from the participants’ perspectives.

Due to the lockdown situation due to the first COVID-19 pandemic established by the Spanish Government [28], interviews were conducted via a private video chat room using the Zoom videoconferencing platform (www.zoom.us, San Diego, CA, USA) [29]. All of the interview procedure using the zoom videoconferencing platform has been previously described [13,14]. All interviews were audio- and video-recorded, recording a total of 1434 min of interviews overall (average of 47.8 ± 12.8 min each interview).

### 2.4. Data Analysis 

Afterwards, full literal transcription of each interview and the researchers’ field notes were collated to perform a thematic inductive qualitative analysis [22,26,30]. Table 2 shows the analysis and trustworthiness criteria used in this study.

## 3. Results

Thirty PTs (19 females, mean age: 41 ± 6 years) working at the Rehabilitation/Physical Therapy Services of 11 public hospitals of Madrid (Spain) during the first wave of the COVID-19 pandemic with a mean of 20 ± 7 years of clinical experience were enrolled. Twelve PTs had suffered COVID-19, five had family members infected with COVID-19, and two had experienced the death of a loved one due to COVID-19.

**Figure 1:**
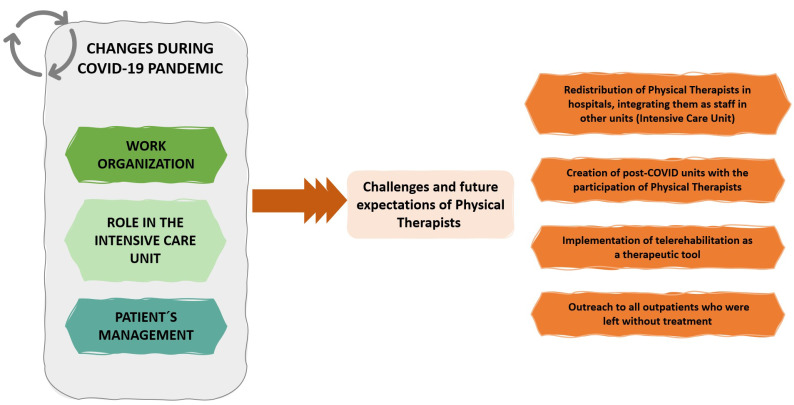
Schematic representations about the changes experienced during the pandemic and changes described as potential new challenges for the physical therapy profession in the future.

Data analysis revealed 1110 codes, and four main themes were identified (Table 3). Throughout the first wave of the pandemic, many changes affecting the work and the role of PTs in the hospitals occurred. These changes took place at the organizational level and affected the distribution of PTs in the hospital, the role of PTs in frontline units such as ICUs, and the management of outpatients both at the beginning of the pandemic, and after discharge from the ICU and acute care units. These changes were described as potential new challenges for the physical therapy profession in the future (Figure 1). We reported some of the participants’ narratives taken directly from the interviews regarding the four emerging themes.

### 3.1. Main Theme: Work Organization during the COVID-19 Pandemic

Physiotherapists described how their work had changed during the pandemic, and how they had been integrated into work teams in other units.

#### 3.1.1. A before and after COVID-19

Prior to the pandemic, the activity of all PTs was centered in the rehabilitation service. On reflection, PTs felt that they worked too independently, had almost no interaction with other healthcare professionals, and did not seem to belong to the hospital. They used terms as “routine”, “programmed”, “limited”, “bubble”, “between walls”, “disconnected from the hospital”, “staying in their comfort zone”, for defining work before COVID-19.

*“Physios do not usually ask to other professionals about a patient, we go to the patient, we apply our techniques and go back to the rehabilitation service.” (p. 17)*

At the beginning of the COVID-19 pandemic, PTs felt that they were working in a sort of “no man’s land”, they felt “out of place”, and useless; they were unaware of how to contribute and how they help. However, during the first weeks, PTs discovered new areas, outside of rehabilitation services, of work at the hospital, where they had the opportunity to change their work organization and were integrated into other healthcare professional teams. They defined this as “a professional leap”:

*“I wish the pandemic had never happened, but it has forced PTs to change their perspective, their role in the hospital. The pandemic forced us to think differently and to perceive our profession differently. It has opened up a world of possibilities” (p. 11)*

#### 3.1.2. The Meaning of Teamwork

The inclusion of the PTs into the teams of other units meant (a) being involved in the decision-making process; (b) transversality among professionals, absence of hierarchies; (c) a great interaction and communication among team members; (d) a high degree of cohesion, defined by the PTs as a strong connection between all healthcare members, based on solidarity, mutual support, an attitude of consensus, and acceptance of any idea or initiative that could save human lives.

*“The hospital has a previous hierarchy, and suddenly it disappears. COVID-19 makes these divisions disappear. Everything becomes transversal, everyone helps. A medical doctor could be putting someone in a prone position or helping out with someone’s toileting” (p. 29)*

PTs explained that they worked as interdisciplinary teams, characterized by constant communication, integration of the knowledge of different professionals, and participation in decision-making among all team members. They compared this to their experience in multi-professional teams, before the pandemic, where each professional carried out the interventions separately, without sharing decision-making with the team members.

*“… the fact that several professionals may be working on the same patient does not mean that it is an interdisciplinary team. If each professional works in their own field, without consulting others, enclosed in their own area of knowledge, nothing is achieved. We have to learn a great deal from each other” (p. 17)*

Eight PTs described conflicts due to the integration of PTs in other units with COVID-19 patients. Although their incorporation was requested by ICU physicians, the reasons given to prevent their incorporation were that in acute care physical therapy assistance is not justified; since they had no experience with personal protective equipment (PPE), they could contaminate themselves and infect other professionals. 

### 3.2. Main Theme: The Role of Physical Therapists on Intensive Care Units (ICU)

Due to the large-scale provision of ICU beds, it was necessary to create prone teams in all units, and PTs were asked to join the prone and respiratory therapy support teams. All PTs narrated how most of them had no previous experience in respiratory physical therapy or pronation, and had to self-train on their own initiative, using social networks, and digital platforms during the first weeks of the outbreak. No centralized training was provided from the hospital management.

All PTs described that “trial and error method” was used, and those interventions that improved the patient’s condition or delayed their deterioration or death were maintained.

*“The massive use of pronation to improve ventilation was prompted used for saving lives. It was the result of trial and error. Whatever did not work was quickly discarded; whatever succeeded in delaying the patient’s death was applied until it was replaced by something better” (p. 22).*

Despite the benefits of prone positioning, PTs describe this procedure as an aggressive technique, which impacted them emotionally, because of its frequency and associated poor prognostic significance:

*“The prone position “per se” is not so shocking, it’s a postural change. What hits you is when you have seven prone positionings in an award or seeing 25 patients in the prone position each day. Prone positioning means that things did not going well” (p. 15)*

Some PTs reported the expression “*it is necessary to de-prone in order to free this bed*”. This meant that there were patients who failed to respond to treatment, and, hence, the prone position was withdrawn because therapeutic possibilities had been exhausted.

*“De-prone to free the bed. To free … to die ... they call it freeing ... it means that the patient was dying, that we can’t do anything more. The objective was to free the bed to try to save the life of the next patient” (p. 17)*

All PTs also helped in the ICU by applying physical respiratory therapy, progressive mobilization, neuromuscular recovery, and pain management until that particular patient was discharged. All PTs reported that their work in the ICU helped to reduce hospital stay and decrease complications due to prolonged immobility.

*“The reason why the PT was incorporated into the ICU was that if the PT improved the patient’s respiratory status so that artificial ventilation was no longer required, the respirator was then transferred to another patient. In this way, a patient who was able to maintain his/her respiratory stability was discharged from the ICU, creating a space the next patient. There was a great need for ventilators and ICU beds.” (p. 22)*

### 3.3. Main Theme: Patient Management

The management of outpatient patients after the outbreak of the pandemic and of COVID-19 patients after discharge from the ICU was also discussed.

#### 3.3.1. Physical Therapy in Outpatients

All PTs recounted how the closure of the rehabilitation service was not immediate. They tried to maintain the activity as much as possible, while trying to avoid the spread of the virus. Progressively, outpatient and home-based rehabilitation settings were discontinued, the duration of treatments was reduced, or in-person treatment was replaced with home exercises.

After closure of rehabilitation services, an attempt was made to maintain treatment and follow-up through videoconferencing, and telephone and exercise videos, however, the redistribution of PTs throughout the entire hospital made this option unfeasible.

*“At the beginning of the outbreak, we tried to monitor our patients, but it was impossible, every day you did something else, and the truth is that we all focused on COVID-19 patients, all efforts were directed to them.” (p. 25)*

All treatments were abruptly stopped, with no alternative. Many PTs feel they abandoned their outpatients:

*“With this pandemic situation, outpatients have been left stranded, untreated, abandoned by all of us. They will be on a waiting list for a long time.” (p. 28)*

#### 3.3.2. Physical Therapy and Post-ICU Patients

All PTs described that COVID-19 patients discharged from the ICU were very fragile, unable to tolerate any type of activity, any movement produced a severe drop in oxygen saturation, and the PT had to monitor all repercussions of any exercise or manipulation.

*“Patients are extremely fragile, they can’t even stand a passive mobilization, they have declined a lot due to long-lasting immobilization. There are patients who have been immobilized between 20 and 40 days.” (p. 24)*

All PTs scheduled frequent rest periods for patient recovery, and as a consequence, for a single session, the PT had to stay with each patient for a prolonged period of time.

*“Everything was extremely slow; you knew when you entered the ward but not when you left.” (p. 9)*

All PTs remarked how the response of COVID-19 patients to the treatment was totally unpredictable, they experienced sudden changes in respiratory frequency and heart rate, extreme exhaustion, sharp drops in oxygen saturation.

*“Apparently, respiratory physiotherapy in COVID-19 patients was no different from other pathologies... But the patients’ response was totally disconcerting, they had exaggerated responses to minimal stimuli or manipulations.” (p. 7)*

The PTs began their work at a motor level, with kinesitherapy. Respiratory therapy was delayed due to patient exhaustion and/or the risk of contagion (due to the generation of aerosols) and the lack of PPE to ensure the protection of the PT. 

All PTs emphasized the collaborative and proactive attitude of COVID-19 patients and their involvement in the treatment. Patients were eager to work, so as not to delay their recovery.

*“The patients are very eager to recover. They are super active and cooperative. They only have one thing on their minds, to recover as soon as possible and return with their families, and they don’t want to waste this opportunity.” (p. 29)*

### 3.4. Main Theme: Challenges and Future Expectations for Physical Therapy

The following proposals were proposed by PTs to improve physical therapy services in hospitals based on the challenges they faced:

(a) The creation of post-COVID units with the integration of PTs, where the sequelae of the virus are studied and treated, and where the PT should have a relevant role in the recovery of neuromotor and respiratory functions, pain, and health education in case of chronification.
  *“With everything we have seen, post-COVID units are not an option, they must be a priority. We are now with acute treatment, vaccines... The sequalae, chronicity and disability will come after. We’re going to be overwhelmed. The physio must be there.” (p. 24)*
(b) The redistribution of PTs in hospitals, integrating PTs as a staff in other units where they were not included before, e.g., ICU. The PT’s work during the first pandemic has shown the effect of physical therapy in acute units outside the rehabilitation services, improving patient recovery and decreasing complications and sequelae.
  *“We have demonstrated that physical therapy can be performed in acute units. We have a role; we can contribute and greatly improve patient’s condition with early physical therapy. We have to leave the rehabilitation service.” (p. 30)*
(c) The implementation of telerehabilitation as a therapeutic tool, providing resources and specific training. In addition, to create hospital physical therapy support units for primary care. Due to COVID-19, patients will present states of maximum fragility and neurological and respiratory sequelae that will require treatment at home and in the general community.
(d) Outreach to all outpatients who were without rehabilitation treatment, by creating a specific consultation/unit for these patients and stating an evaluation of their condition and reincorporation into physical therapy treatment.

## 4. Discussion

This qualitive study describes how a group of Spanish PTs from public hospitals experienced their professional duties and roles during the first outbreak of the COVID-19 pandemic. The main themes that emerged from the interviews emphasized the professional organization, the experience shared with the patients, the unexpected insertion in ICU teams and the future professional expectations while they were experiencing the COVID-19 pandemic. Table 4 summarizes the process used for obtaining these results. 

The reported change in patient care in the hospitals was not a reality exclusive to Spain. Hospital services were reorganized and focused their material and personnel resources on acute care during the first outbreak of the COVID-19 pandemic in several countries such as Italy, China, or the United States of America (USA) [8]. This led to the closure of rehabilitation services and the redistribution of PTs to other units [6,8,13,31]. Negrinit et al. [10] reported that stopping of admissions to rehabilitation, early discharge, and reduction of activities involved 194,800 inpatients in 10 countries. Additionally, outpatient activities stopped for 87%, involving 318,000 patients/day in Italy, Belgium, and the United Kingdom (UK), leading to an estimate range of 1.3–2.2 million outpatients in Europe [10].

The guidelines of The Spanish Society of Physical Medicine and Rehabilitation and of the Spanish Society of Intensive, Critical Medicine and Coronary Units included the need for rehabilitation treatment in Post-Intensive Care Syndrome (PICS) [11,12] and described the criteria for early mobilization and respiratory rehabilitation in patients with COVID-19 [32]. However, despite these recommendations, our participants experienced conflicts with their rehabilitation services in order to be integrated into the ICUs.

The presence of these conflicts in the first weeks of the first outbreak in relation to the presence of PTs in units such as the ICU could be justified by the contrast between the sense of duty that led PTs to voluntarily report to acute care [14,17] and the concern in rehabilitation services to find a balance between providing rehabilitation care to as many patients as possible, avoiding the contagion of patients at risk and protecting rehabilitation professionals, as described in countries such as Italy and Spain [6,8,11,32].

After the initial conflict, our results show that PTs participated in the care of COVID-19 patients who were admitted to acute and ICU units, by supporting professionals (prone teams) and subsequently applying physical and respiratory therapy in patients extubated and discharged from ICU. Previous studies [14,17,31] show great variability in the role of PTs in other units during the first outbreak, including screening, triage, supporting safe early discharge, and supporting other hospital staff (e.g., physicians, nurses, social workers, pharmacists) where resources were scarce. Additionally, the within-hospital roles for PTs include multiple settings, including emergency department (e.g., management of musculoskeletal concerns to assist with patient flow) and ICUs (e.g., prone teams, preventing/addressing effects of immobility) [33,34,35,36].

Our results are consistent with previous studies [13,17] showing that once incorporated into ICUs and acute care units, PTs had the feeling of being lost, of not being sufficiently useful or also that they wanted to contribute more. In fact, their training, mainly oriented to rehabilitation and comprehensive recovery of outpatients, could place them in a more vulnerable position in relation to the emotional impact of working in first-line units [14], without having experience and/or training to deal with critical events associated with a situation such as death, disaster situations, and moral dilemmas and conflicts during the COVID-19 pandemic [13,14,17]. Our results also showed that PTs had to self-train to adapt to the demands of the pandemic care. Pedersini et al. [35] specifically reported the creation of a “physical therapy task force” to help therapists improve skills that may be required to care for patients with COVID-19.

The PTs interviewed described how they became integrated into the teams of other units, highlighting the teamwork and the interdisciplinary nature of the work. According to Wittmeier et al. [31], the PT presents various competencies within an inter-professional team-based practice during a pandemic scenario: (a) individual professional competencies (PT knowledge and skills); (b) common competencies (e.g., patient positioning); (c) inter-professional collaboration competencies (e.g., communication skills). This contrasts with The Spanish Society of Physical Medicine and Rehabilitation (SERMEF), which promotes the transition to real multi-professional rehabilitation teams based on a bio–psycho–social model, although oriented to professionals that can help boost the technology applications (bioengineers) and, most importantly, dealing with more robustness with the psychosocial aspects of rehabilitation patients (psychologists and social workers among others) [11].

There appears to be a lack of understanding among other healthcare professionals, regarding the knowledge and skills possessed by a PT that are relevant in acute care and disaster situations [36,37,38,39]. The PT plays a key role in early patient mobilization, avoidance of deconditioning, promotes patient safety, and assists in the establishment of an optimal plan of care and an appropriate discharge setting for each patient [31,38]. Moreover, physical therapy roles that are important during a disaster situation also include acute care skills such as early immobilization and respiratory care [31,37,40]. The lack of understanding of the role of PT in acute units can be explained by the lack of support from the institutions and their managers, the lack of a clear outline of the role of physical therapy, and the lack of information and education of other healthcare professionals about PTs and their role [36,37,39]. Li et al. [41] described the effect of including PTs into ICUs to assist in the management of patients requiring mechanical ventilation during the COVID-19 pandemic. Physical therapy interventions included body positioning, airway clearance techniques, oscillatory positive end-expiratory pressure, inspiratory muscle training, and thorax mobility exercises. These authors concluded that although the respiratory and physical functions of some patients remained poor at ICU discharge, the interventions by PTs were safe and appeared to be associated with an improvement in respiratory and physical function in those COVID-19 patients who were in the ICU.

The presence of post-acute sequelae and disability due to COVID-19 will be also an opportunity for PTs to lead and/or participate in post-COVID-19 units. The most prevalent sequelae of severe COVID-19 will affect the respiratory, cognitive, central and peripheral nervous systems, and may include deconditioning, critical-illness related myopathy and neuropathy, dysphagia, joint stiffness and pain [8]. The presence of long-COVID symptoms leads to varying degrees of disability [42]. Of particular relevance for PT will be the management of musculoskeletal pain as long-term post-COVID sequelae [43]. A definition of post-COVID symptoms is currently under study to enable profiled care for COVID-19 survivors [42,44]. 

Another important patient group to consider is that one consisting of disabled patients who did not receive treatment or whose treatment was discontinued due to the pandemic. In Europe, up to 2.2 million people with disabilities suffered collateral damage every day as a result of the closure of services due to COVID-19 [10]. Despite suspending treatments, the rehabilitation services established priority criteria in the different settings, stressing the need to use alternative treatment modalities such as telerehabilitation or telephone services during the worldwide lockdown in the absence of face-to-face treatment [6,7,8,45,46,47,48]. Our results show that this follow-up was not carried out by most of the PTs included in this study, although they highlighted telerehabilitation as a potential change in the future of the profession. There are barriers in the application of telerehabilitation, in the attitude of professionals and in the available resources allocated to its implementation. Among professionals’ beliefs dealing with musculoskeletal conditions, Malliaras et al. [47] reported that PTs felt they lacked adequate training to deliver telehealth services and that telehealth was less effective than face-to-face care and undervalued by patients themselves. This study also identified barriers affecting clinicians’ perceptions, related to risk assessment or safety, the patient–therapist relationships, environmental barriers (e.g., the space for patient treatment and application of health services), physical distance (lack of physical interaction), management delivery (hands-on), lack of hands-on (impact on assessment quality/accuracy and impact on management quality/accuracy—patients’ perspectives) [47]. Conversely, the facilitators for telerehabilitation include less travel time for patients, ease of use of the system and a shorter waiting period for patients [48]. Taito et al. [49] in their paper on telerehabilitation in patients with respiratory disease during the pandemic, described how the reported number of adverse events was low and the average treatment compliance rate was greater than 70%. In the Netherlands, Bos et al. [48] showed positive results after using telemedicine for patients with rheumatic and musculoskeletal diseases during the COVID-19 pandemic. 

Undoubtedly, if telerehabilitation is increasingly implemented in daily practice of physical therapy, there should be clear and practical recommendations for telerehabilitation to improve the use of technology as an alternative mode of delivering physical therapy [50].

### Strengths and Limitations

One of the strengths of this study is that this sample provides an in-depth insight (i.e., 99% of potential emerging themes) into the participants’ experiences [16,20]. We should consider the fact that some participants were infected by COVID-19, which may have influenced the results; nevertheless, the use of a question guide helped to orient and focus the responses of all participants, to answer the objectives of the present study. In addition, the professional role of the participants is that of PTs working in public hospitals in Spain. These findings therefore do not reflect the role of PTs worldwide during the COVID-19 pandemic, as the physical therapy profession and legal competences are widely variable from one country to another. The perspectives of PTs who traditionally work in an ICU team would most likely be different.

## 5. Conclusions

This study provided an in-depth understanding of the experience of PTs attending COVID-19 patients during the first outbreak regarding new duties and organization of their work, their integration in prone teams, post-ICU discharge physical therapy, and the overall challenges for the physical therapy profession. These results may help to develop new training programs for teaching physical therapy in the context of future pandemics, based on our experience with the COVID-19 pandemic [51].

## Figures and Tables

**Table 1:** Semi-structured question guide.

Issue Studies	Questions
Team healthcare during the COVID-19 outbreak	In which hospital services have you been working during the COVID-19 pandemic? What were your role and functions? How was it working in the healthcare team? What was the most relevant part of that experience for you?
Technical and organizational changes in physical therapy during the COVID-19 pandemic	How do you think the pandemic has changed the way physical therapists work and/or are organized in hospitals? What changes have been the most relevant to you? How did you experience your first day in other services (different than rehabilitation), such as the intensive care unit, emergency department, etc.?
Patient management during the COVID-19 outbreak	How were outpatients in the rehabilitation service cared for during the pandemic? How was the monitoring and management of outpatients who attended rehabilitation prior to the COVID-19 pandemic carried out?
The future of physical therapy, and challenges during the COVID-19 outbreak	Do you think the pandemic has changed your profession (physical therapy)? What should physical therapy learn from the COVID-19 pandemic? What expectations do you have for physical therapy? What would you change about your professional performance during the COVID-19 pandemic? What consequences after COVID-19 do you think will reconfigure the work of the physical therapist? What priorities in rehabilitation will appear after surviving COVID-19?

**Table 2:** Analysis and trustworthiness criteria [22,26,30].

Thematic analysis	Step 1: Identifying the most descriptive content (codes). Step 2: Subsequently identify the categories (code groups). Categories included similar points or content that allowed the emergence of the topics that described the participants’ experiences. Step 3: Subsequently, joint meetings were held to combine the results of the analysis. In the case of differences in opinion, theme identification was performed based on consensus among the research team members. This thematic analysis process was separately done upon the semi-structured interviews. No qualitative software was used to analyze the data.
Trustworthiness method	Credibility using cross-triangulation by the researchers, participant triangulation, triangulation of methods of data collection, participant validation. Transferability using in-depth descriptions of the study performed. Dependability using audit by an external researcher. Confirmability using researcher reflexivity.

**Table 3:** Themes and categories identified.

Theme	Category
Work Organization during the COVID-19 Pandemic	A before and after. The meaning of teamwork
The Role of Physical Therapists on Intensive Care Units (ICU)	
Patient Management	Physical therapy in outpatients. Physical therapy and post-ICU patients.
Challenges and Future Expectations of Physical Therapy	

**Table 4:** Summary of methods and results.

Research question	
What is the perspective and experience of physical therapists with the organization of their work during the COVID-19 pandemic? What was their role in intensive care units (ICU) and in the management of COVID-19 survivors? What changes might the pandemic mean for the future physical therapist professional?	
Objectives	
The purpose of this study was to explore the experiences and perspectives of Spanish physical therapists regarding, (a) the organization of their work during the first wave of the COVID-19 pandemic; (b) their role in ICUs; (c) management of COVID-19 survivors; (d) future challenges identified for the physical therapy profession.	
Methods	
Study Design	A qualitative exploratory study based on an interpretive framework was conducted.
Ethics	Received approval by the Local Ethics Committee of Universidad Rey Juan Carlos (URJC 1905202011920).
Research Team	Five researchers (three women) participated in this study, including one anthropological nurse (DPC) and four physical therapists (CFdlP, LLF, MPC, ALLR). All researchers have experience in research in health sciences and none were involved in related clinical activity associated with the participants.
Participants, Context, and Sampling Strategies	*Participants:* 30 physical therapists were recruited. *Sampling Strategies*: A purposeful sampling and snowball sampling were used. *Inclusion criteria* were being a physical therapist working on the frontline (direct contact with COVID-19 patients) in public hospitals during the first wave of the COVID-19 pandemic. *Context:* Eleven public hospitals in Madrid (Spain).
Data Collection	Based on the qualitative exploratory design, in-depth interviews were used as the main data collection tool.
Data Analysis	Thematic inductive qualitative analysis was used.
Rigor	Trustworthiness method using Credibility, Transferability, Dependability and Confirmability criteria was used.
Results	
We obtained four themes: Theme 1. Work Organization during the COVID-19 Pandemic, with two main categories; A before and after, and, The meaning of teamwork. Theme 2. The Role of Physical Therapists on Intensive Care Units (ICU). Theme 3. Patient Management with two main categories; Physical therapy in outpatients and Physical therapy and post-ICU patients. Theme 4. Challenges and Future Expectations of Physical Therapy.	

## Data Availability

The data presented in this study are available on request from the corresponding author. The data are not publicly available due to legal and ethics reasons.
